# The Mediterranean Diet: An Update of the Clinical Trials

**DOI:** 10.3390/nu14142956

**Published:** 2022-07-19

**Authors:** Mauro Finicelli, Anna Di Salle, Umberto Galderisi, Gianfranco Peluso

**Affiliations:** 1 Research Institute on Terrestrial Ecosystems (IRET), National Research Council of Italy (CNR), Via Pietro Castellino 111, 80131 Naples, Italy; anna.disalle@cnr.it; 2 Department of Experimental Medicine, University of Campania “Luigi Vanvitelli”, Via Santa Maria di Costantinopoli 16, 80138 Naples, Italy; umberto.galderisi@unicampania.it; 3 Faculty of Medicine and Surgery, Saint Camillus International University of Health Sciences, Via di Sant’Alessandro 8, 00131 Rome, Italy

**Keywords:** Mediterranean Diet, clinical trials, inflammation, oxidative stress, cardiovascular disease, metabolic diseases, cancer, diabetes

## Abstract

The Mediterranean Diet (MedDiet) is a term used to identify a dietary pattern originating from the unique multi-millennial interplay between natural food resources and the eating practices of people living in the Mediterranean basin. Scientific evidence has described the healthy properties of the MedDiet and its beneficial role in several pathological conditions. Nevertheless, current socio-economic trends have moved people away from this healthy lifestyle. Thus, clinical and biological evidence supporting the benefits of the MedDiet is needed to overcome these limitations. Clinical nutrition research examines the effects of dietary interventions on biological or health-related outcomes in a determined study population. The evidence produced by these studies is useful for dietary guidance and public health messaging. We provided an update of the clinical trials registered on the database clinicaltrials.gov evaluating the effects of the MedDiet on health and specific diseases. Our findings revealed an increased number of clinical trials in the last decade and found that most disease-related studies focused on cardiovascular diseases, metabolic diseases, and cancer. The majority of MedDiet’s beneficial effects could be primarily related to its anti-inflammatory and anti-oxidant properties as well as the effectiveness of this dietary pattern in controlling waist circumference and obesity. Moreover, strict and long-lasting adherence to the MedDiet as well as the beneficial effects of specific components (e.g., olive oil or its polyphenols) seem to emerge as useful insights for interventional improvements. These findings present further insights into the MedDiet’s resources and how it could strengthen overall public health.

## 1. Introduction

The Mediterranean Diet (MedDiet) is a term used to identify a dietary pattern that has caught the attention of clinicians and researchers worldwide. The MedDiet originated from the unique multi-millennial interplay between natural food resources and the eating practices of people living in the Mediterranean basin [1]. It has been widely acknowledged that the MedDiet encompasses many aspects beyond nutritional behavior, including social, cultural, economic, and environmental features. The respect for seasonality, biodiversity, and food varieties, preferring local and fresh products, is a cornerstone of the MedDiet. The association of these cultural and nutritional features with physical activity is woven into the MedDiet model, making it widely considered to be a healthy lifestyle rather than a dietary pattern [2]. UNESCO considers the MedDiet an intangible cultural heritage, given the responsible interactions between agricultural and dietary practices and the environment [3].

Scientific evidence of the MedDiet’s beneficial impacts on human health was first posed for cardiovascular disease. In post-war Europe, the American scientist Ancel Keys studied how famine induced a decrease in coronary attacks and proposed an association between the reduction in high-fat and high-calorie diets and coronary outcomes. At the same time, Keys observed a higher incidence of heart attacks among middle-aged businessmen in the United States. These findings suggested the possible influence of dietary habits on cardiovascular risks and became the basis for Keys and colleagues’ seminal “Seven Country Study” [1,4,5]. This study reported cardiovascular disease incidences and mortality data in seven different Mediterranean region countries [3,6]. Despite this promising evidence, the MedDiet’s positive effect on human health was not globally recognized until the 1990s [7]. Over the years, a large body of evidence has sustained the health benefits of the MedDiet for cardiovascular disease (CVD), type 2 diabetes (T2D), metabolic syndrome (MetS), obesity, and cancer [3,8,9]. Nevertheless, the scientific evidence for each outcome is variable, and more studies are required to understand the influential role of the MedDiet better. In this context, the most useful approach involves intervention studies, given their compelling evidence [10]. The health benefits associated with the MedDiet are undervalued by current socio-economic trends that move people away from this healthy lifestyle. Unfortunately, this trend affects MedDiet homeland countries where younger generations often prefer western-style food consumption. This paradox must be changed to protect the precious heritage passed on by the MedDiet’s positive impacts on health and sustainability [1,2]. Medical evidence is the keystone to sustaining and protecting the MedDiet and its clinical implications. 

This review updates the MedDiet-based clinical trials and addresses current knowledge on the primary diseases, topics, and outcomes impacted by this diet. The scientific evidence emanating from observational and intervention clinical studies provides valuable insights into the MedDiet and robust proof of its positive health benefits [11]. These findings present further insights into the MedDiet’s resources and how it could strengthen public health. 

## 2. The MedDiet: Definition and Composition

**Figure 1:**
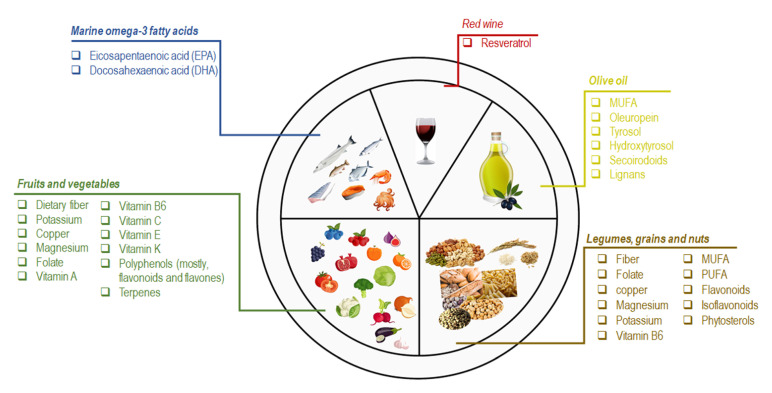
Schematic representation of the nutritional and bioactive characteristics of the principal components of the MedDiet.

The term MedDiet commonly refers to the dietary pattern of the people living on the Mediterranean Sea coast, in particular Greece, southern Italy, and southern Europe [10,12]. Although these Mediterranean countries show some eating habit differences, the common features characterizing the MedDiet are defined as (a) daily consumption of non-refined cereals and other products (e.g., whole grain bread, whole grain pasta, and brown rice), fresh fruits, vegetables, nuts, and low-fat dairy products; (b) olive oil as the principal source of lipids; (c) moderate intake of alcohol, preferably red wine, with meals; (d) moderate consumption of fish, poultry, potatoes, eggs, and sweets; (e) monthly consumption of red meat; and (f) regular physical activity [8,12,13]. Besides these characteristics, the beneficial impact of the bioactive molecules in the essential individual components of the MedDiet has also been considered. In this section we provide a brief overview of the principal elements and clinical implications recognized for the significant features of the MedDiet (Figure 1). 

Marine omega-3 fatty acids are the most important bioactive molecules in fish and seafood consumed in the MedDiet (e.g., sardines, mackerel, mussels, octopus, salmon, squid, and tuna). Eicosapentaenoic acid (EPA) and docosahexaenoic acid (DHA) are the major n-3 fatty acids recognized for their cardioprotective effects [14]. In a meta-analysis of a randomized controlled trial (RCT), EPA and DHA supplementation reduced the risk of coronary heart disease (CHD) in higher-risk populations [15]. Analogously, the high intake of fish is accountable for the beneficial effects on HDL cholesterol and triglycerides levels [16].

Olive oil (OO), and especially extra virgin olive oil (EVOO), is the primary source of fat in the MedDiet. Monosaturated fatty acids (MUFAs) are the most representative OO fatty acids (FA), ranging from 55–83% of the total FA content [17]. Saturated fatty acids (e.g., palmitate) impair mitochondrial respiration by causing an increase in both total cellular ROS and mitochondrial ROS, contrary to MUFAs, such as oleate from OO [18,19]. Nevertheless, controversies still exist on the beneficial effect of MUFA on human health. On the other hand, much attention must be given to the lesser components of OO (2% of the total weight), consisting of polyphenols or other secondary plant metabolites (e.g., oleuropein, tyrosol, hydroxytyrosol, secoirodoids, and lignans) [20,21]. Overall, these molecules contribute to the beneficial effects of OO and EVOO on human health. A meta-analysis of 32 observational studies revealed that OO consumption decreased the risk of stroke, CHD, and diabetes and improved some metabolic and inflammatory biomarkers [22,23,24]. The consumption of OO enriched in phenolic compounds seemed to reduce the urinary levels of DNA oxidation (8-OHdG) and inflammatory (IL-8, TNF-α) markers in an opportune population cohort [25]. The regular intake of 15 mg/day of HTyr resulted in modification of body composition parameters and modulated the antioxidant profile, the expression of inflammation and oxidative stress-related genes in atherosclerosis [26].

Fruit and vegetable consumption is emphasized in the MedDiet. Oranges, pomegranates, berries, figs, and grapes are the most frequently consumed fruits and a source of dietary fiber, potassium, vitamin C, polyphenols (mostly flavones), and terpenes [8]. Vegetables are mainly seasonal and field-grown. The most representative vegetables are fresh greens, tomatoes, eggplants, cabbages, radishes, garlic, onions, spinach, and lettuce. Although these foods are an essential source of nutrients (e.g., dietary fiber, potassium, copper, magnesium, folate, vitamin-A, -B6, -C, -E, -K), the phenolic compounds (mainly flavonoids) are the most important bioactive molecules [27,28]. Several studies demonstrated that high consumption of vegetables or fruit resulted in lower risk for all-cause mortality, CHD, stroke, T2D, colon rectal cancer (CRC), and adiposity [16,29,30,31,32,33].

Legumes, grains, and nuts are regularly consumed in the MedDiet. Grains appear both as a single food (e.g., rice and oatmeal) and as ingredients of processed foods (e.g., bread, pasta, cereal, and crackers). Common MedDiet nuts include almonds, hazelnuts, walnuts, and pistachios. Among legumes, the most representative is lentils, beans, and chickpeas. Overall, these foods are a valuable source of fiber, folate, vitamin B6, magnesium, potassium, and copper [27,30]. In particular, nut intake is crucial because these foods are unique for their MUFAs and polyunsaturated FA (PUFAs) content, especially linoleic, linolenic acid, phenols, flavonoids, isoflavonoids, and phytosterols [12,34]. The beneficial effect of nut consumption primarily impacts the incidence of CVD, diabetes, and MetS [35,36,37]. The beneficial effects of legumes and grains on CVD, body weight, and cholesterol (total and LDL-C) have also been described. Of note, grain consumption has also been associated with a lower risk of T2B and CRC [16,29,30,31,32,33].

Red wine is routinely consumed MedDiet meals. Resveratrol is the most abundant polyphenol in red wine. Besides the acknowledged effect of this molecule on several chronic diseases (e.g., cancer, myocardial infarction, and brain disorders), evidence supports its role in protecting against the development of some MetS features [12,38]. 

Although this body of evidence demonstrates the role of nutrients and foods in the pathophysiology of numerous diseases, the mechanisms by which the MedDiet exerts its favorable effects are not fully understood. However, the five most important influences induced by adherence to the MedDiet can be summarized as (a) lipid-lowering effect, (b) anti-oxidative and anti-inflammatory action, (c) modification of key molecules (hormones and growth factors) involved in the pathogenesis of cancer, (d) inhibition of nutrient-sensing pathways, and (e) gut microbiota-mediated production of metabolites influencing metabolic health [39]. The current research focuses on the MedDiet pattern as a whole. The overall combination of MedDiet foods and their components’ additive or synergistic effects seems to provide more consensus regarding health benefits [3]. Moreover, food production, consumption, cooking techniques, and eating behaviors are also emerging as innovative variables used to assess the beneficial effects of the MedDiet [40]. Accordingly, research must go beyond the common links between foods and nutrients and consider other aspects characterizing the MedDiet as a healthy lifestyle. 

## 3. MedDiet and Immunity 

The relationship between diet and immune response is a very close concept, although their interaction is extremely complex. The nutrition status as well as the specific micronutrients can influence the function of the immune system at various level up to the microbiota [41,42]. For example, FAs impact on macrophage function, influencing the release of immunomodulatory factors (i.e., cytokine and chemokines) and the polarization phenotype [43,44]. Again, undernutrition can lead to a lower efficacy of the immune system, by compromising the components needed to drive an effective response [41]. 

MedDiet is associable with immunomodulatory properties and a reduced incidence of inflammation, because of the effects played by some components, such as MUFA, vitamins, polyphenols, minerals and micronutrients [41,45].

Oleocantal, an olive oil polyphenol, contributes to inhibiting the activity of cyclooxygenase 1 and 2 (COX1 and 2), key enzymes driving the inflammatory process, as well as hampering the lipopolysaccharides-mediated up-regulation of pro-inflammatory factors, such as IL-1β, IL-6, TNF-α [46,47]. Ω-3 PUFAs are abundant in MedDiet and provide anti-inflammatory and immunomodulatory effect by influencing the function of immune system. These molecules down-regulate the expression of pro-inflammatory factors, such as IL-1β, IL-6, TNF-α, VCAM-1, MCP-1, reduce the levels of ROS and nitrogen species with a concomitant increase of anti-inflammatory cytokines, as IL-10 [48,49,50]. 

The high intake of fiber also characterizes the MedDiet pattern and provides essential benefits to the intestinal microbiota by modulating its composition and favoring the release of metabolites, such as the short-chain fatty acids (e.g., acetate, propionate and butyrate), that regulate the immune functions [41]. For example, butyrate exerts anti-inflammatory effects by reducing the production of IL-1β, TNF-α, NF-kB and IL-12 [51]. The adherence to MedDiet also contributes to the reestablishment of microbiota eubiosis, by favoring the colonization of Bacteroidetes and certain beneficial Clostridium groups and decreasing Proteobacteria and Bacillaceae phyla [52]. Of note, MedDiet seems to be effective in attenuating the pathological microbiota status, by evoking the rise in favorable strains as well as their metabolites ‘production. This is an intriguing finding, given the speculations about the interventional effect of this dietary pattern in mediating the local and systemic response by influencing the microbiota [52,53].

Moreover, MedDiet and its components provide further implication in protecting and mitigating against the major determinants of the immune competence, including stress and pollution. The anti-inflammatory and anti-oxidant potential of this dietary pattern seem to cope with the harmful effect due to exposition to air pollution [54,55,56].

## 4. The MedDiet in Clinical Trials: An Update

Clinical nutrition research aims to examine the effects of dietary interventions on biological or health-related outcomes in a determined study population. This approach is beneficial in the nutritional science field because the evidence produced is pivotal for dietary guidance and public health messaging [57,58]. Thus, an overview of MedDiet clinical trials could be useful in providing state-of-the-art clinical research focused on this dietary pattern and its primary outcomes. This review updates the current MedDiet clinical trials and those where MedDiet adherence and/or the contribution of one or more of its main foods is/are one of the measured outcomes by consulting the public web archive of ClinicalTrials.gov (https://clinicaltrials.gov/; updated on 31 January 2022). This is a public database provided by the U.S. National Library of Medicine that includes privately and publicly funded clinical studies conducted worldwide. This research used the keyword “Mediterranean Diet” to generate a list of 438 clinical trials (updated on 31 January 2022). It further filtered the studies by focusing on “completed” studies. Completed studies refer to the concluded clinical trials where participants finished all the observational steps. Accordingly, 198 completed studies were considered for further analysis. Thirteen studies were eliminated (i.e., NCT02710513, NCT02160496, NCT02767102, NCT03660579, NCT03911843, NCT00433082, NCT01788670, NCT02202863, NCT04191525, NCT01620398, NCT04436614, NCT03740295, and NCT02099968) because they fell into one of the following cutoffs: (a) studies not focusing on the MedDiet, (b) MedDiet did not appear in the study outcomes, and (c) MedDiet appeared only in the study background. For example, study NCT04191525 aimed to characterize the efficacy of treatment with probiotics in patients with T2D at different stages of evolution; thus, a MedDiet contribution was not strictly evident. Analogously, study NCT03660579 examined the effect of habitual and moderate consumption of beer on physical fitness, body composition, psychokinetic and cognitive abilities, and psychological status in sedentary healthy adults; adherence to the MedDiet was only a marginal outcome. Again, clinical trial NCT01788670 focused on the contribution of ethanol to hydroxytyrosol formation, and the term MedDiet was only compared in the background. Another study (NCT00433082) was performed to evaluate the effects of Ramadan fasting in MetS, and the MedDiet was only mentioned in the description paragraph. 

**Figure 2:**
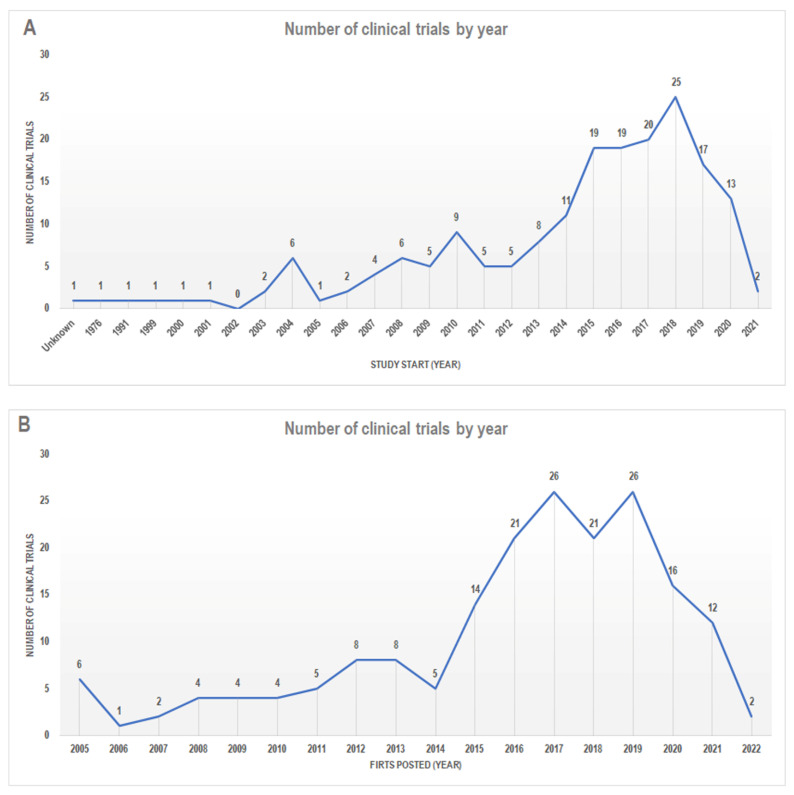
The number of clinical trials classified according to (**A**) “Study Start” and (**B**) “First posted”. Data from http://www.clinicaltrials.gov/ (accessed on 31 January 2022).

Thus, only the remaining 185 studies were ultimately considered. First, we categorized the studies according to the “study start date”. This parameter is supplied by ClinicalTrials.gov and provides indications about the date the first participant is enrolled in a clinical study or the estimated date that the researchers pose as the study start date (https://clinicaltrials.gov; accessed on 31 January 2022). Accordingly, we found that the data from these 185 studies ranged from 1976 to 2021 (only one study has an “unknown” date start) (Figure 2A). Some studies were retrospective, so we also grouped the studies according to the term “first posted.” This term provided the date on which the study record was first available on ClinicalTrials.gov at the end of National Library of Medicine (NLM) quality control (https://clinicaltrials.gov; accessed on 31 January 2022) (Figure 2B). Interestingly, we observed that the number of clinical trials based on the MedDiet had increased substantially since 2012; this is in line with the growth in quality and evidence about the beneficial effects of the MedDiet that has emerged in the last decade [2]. 

**Figure 3:**
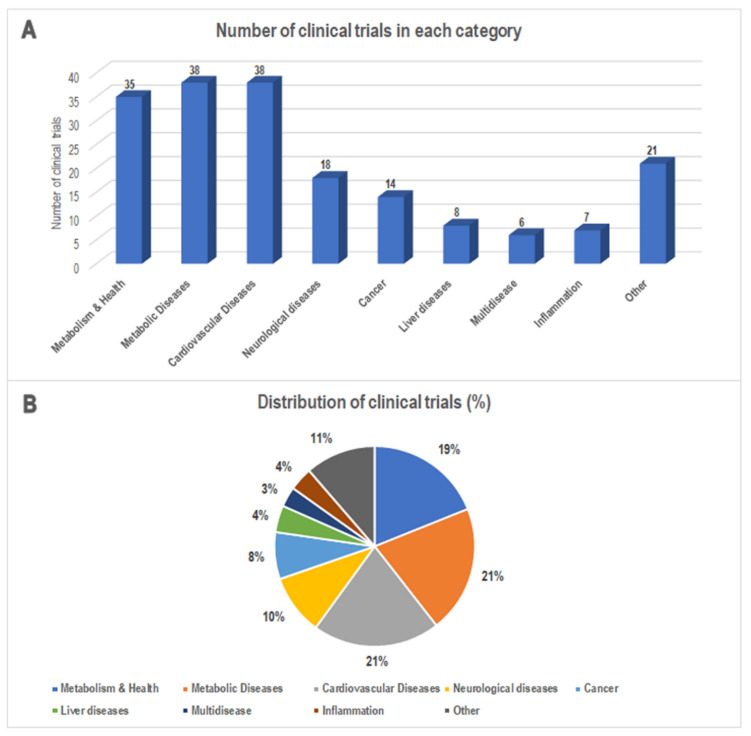
(**A**) The number and (**B**) percentage of MedDiet-based clinical trials classified according to the indications provided by the clinical trials database. Data from http://www.clinicaltrials.gov/ (accessed on 31 January 2022).

We also grouped the 185 studies according to the indications provided in the item “condition” reported on ClinicalTrials.gov (accessed on 31 January 2022) and the main characteristics of each study. This allowed us to classify these studies into nine categories: Metabolism & Health, Metabolic Diseases, Cardiovascular Diseases, Neurological Diseases, Cancer, Liver Diseases, Multidisease, Inflammation, and Other (Figure 3). Of note, the group Metabolism & Health contains the studies in which the MedDiet effect was not strictly evaluated for a particular disease or the study outcomes were verified on healthy volunteers only. We use the term Multidisease to group studies in which the MedDiet contribution was assessed for features of multiple diseases (e.g., cardiometabolic features). Finally, the MedDiet-based studies on a less representative target disease were grouped in the Other group. This categorization let us speculate that most of the disease-related studies fell in the following categories: Cardiovascular Diseases (*n* = 38, 21%), Metabolic Diseases (*n* = 36, 19%), Neurological Diseases (*n* = 18, 10%), and Cancer (*n* = 14, 8%) (Figure 3). 

**Figure 4:**
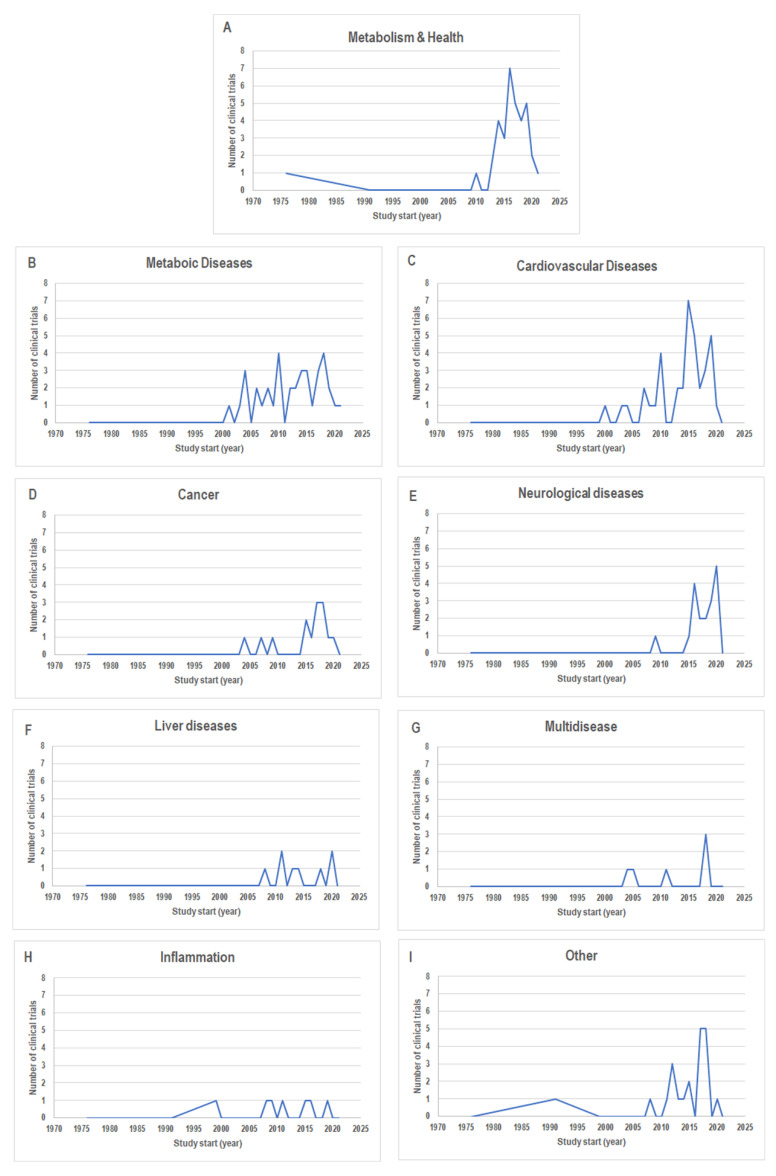
The number of clinical trials for each category classified according to “Study Start” as follow: (**A**) Metabolism & Health; (**B**) Metabolic Diseases; (**C**) Cardiovascular Diseases; (**D**) Cancer; (**E**) Neurological Diseases; (**F**) Liver Diseases; (**G**) Multidisease; (**H**) Inflammation; (**I**) Other. Data from http://www.clinicaltrials.gov/ (accessed on 31 January 2022).

This suggests that these are the most investigated diseases for MedDiet effects. According to the study start date of these four groups, we found that neurological diseases encompassed the more recent data (the most range from 2015 to 2020) (Figure 4E). The Cancer group also contained quite recent data (Figure 4D), while the Cardiovascular Diseases and Metabolic Diseases groups had the widest range of dates (from 2000–2020 and 2001–2021, respectively) (Figure 4B,C).

The graphs showing the number of clinical trials classified according to the study start dates for the other categories are also reported in Figure 4A–I.

**Figure 5:**
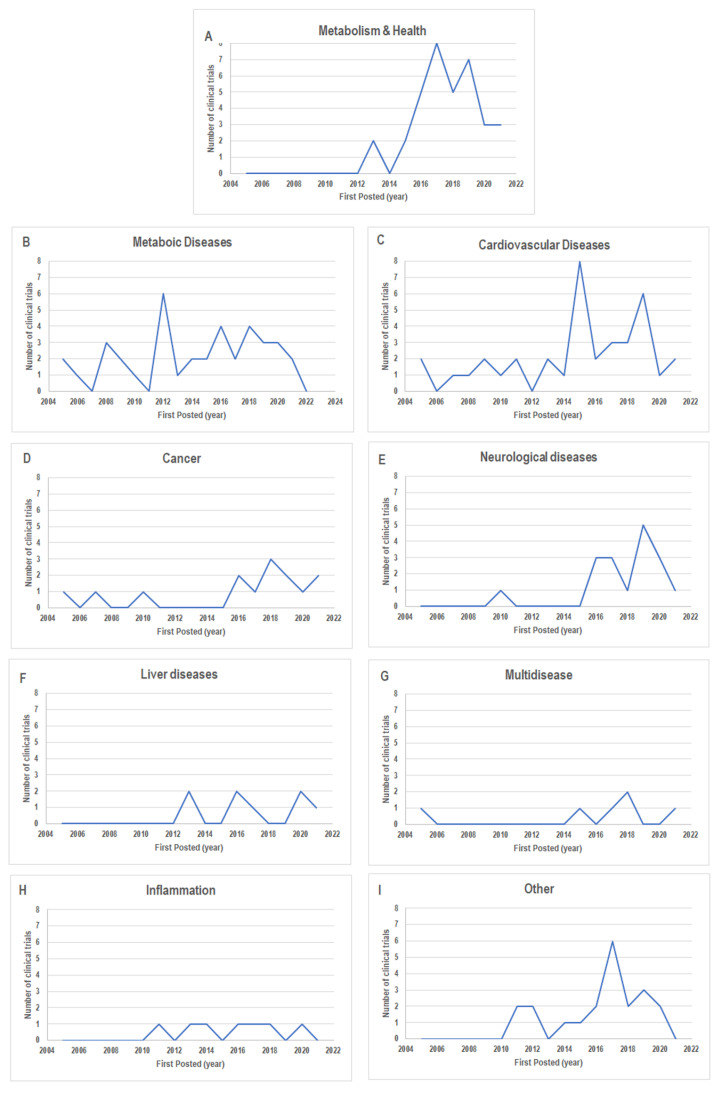
The number of clinical trials for each category classified according to “First posted” as follow: (**A**) Metabolism & Health; (**B**) Metabolic Diseases; (**C**) Cardiovascular Diseases; (**D**) Cancer; (**E**) Neurological Diseases; (**F**) Liver Diseases; (**G**) Multidisease; (**H**) Inflammation; (**I**) Other. Data from http://www.clinicaltrials.gov/ (accessed on 31 January 2022).

The same trends were observed when we looked at the study record (Figure 5A–I). 

## 5. Evidence from the Clinical Trials 

This section provides the results of the clinical trials available on PubMed, among those belonging to the most representative groups: Metabolic Disease, Cardiovascular Disease, and Cancer, as previously described. Neurological Disease was not considered because of the few available studies, probably due to the fact that this group encompasses more recent clinical trials. Briefly, the identifier number of each trial grouped into the categories Metabolic Disease, Cardiovascular Disease, and Cancer was used for Medline and only the published studies were considered.

### 5.1. The MedDiet and Metabolic Diseases

The global rise of energy-dense diets and sedentary lifestyles has led to an increase in overweight and obese subjects [12,59]. Obesity is a central component of MetS and is a risk factor for type 2 diabetes mellitus (T2DM) [59]. Given obesity, MetS and T2DM are interrelated conditions sharing many pathophysiological mechanisms, and their increase and incidence are parallel [59,60]. The adipose tissue disposition triggers a chronic low-grade inflammatory status causative of metabolic disturbances, including insulin resistance and dyslipidemia. These features are also described in MetS; insulin resistance is the core of MetS and dyslipidemia and arterial hypertension, which are, in turn, associated with the risk of T2DM and cardiovascular diseases [61]. 

Lifestyle is a determinant point in treating obesity, MetS, and T2DM. Diet, exercise, and behavior are the three basic components for improving these conditions [60]. Concerning diets, several studies have shown the MedDiet’s beneficial effect on preventing and controlling many metabolic problems, such as obesity, T2DM, MetS, and cardiovascular complications [62,63,64,65].

On these premises, we provide evidence from clinical trials grouped in the category of Metabolic Diseases supporting the MedDiet’s beneficial effects.

Luisi et al. studied (NCT03441802) the effect of a MedDiet enriched in high-quality extra virgin olive oil (HQ-EVOO) in overweight/obese subjects and normal weight controls [66]. The authors tested whether 3 months of an HQ-EVOO-enriched MedDiet could affect inflammation and oxidative stress parameters and gut microbiota composition. Interestingly, in both study groups, this dietary pattern decreased myeloperoxidase (a marker of inflammation and endothelial dysfunction [67]), 8-hydroxy-2-deoxy-guanosine (a characteristic feature of oxidative DNA damage [68]), Tumor necrosis factor-alpha (TNF-α), and Interleukin 6 (IL-6) (molecules exerting a pro-inflammatory effect and impairing insulin receptor signaling [66]). Moreover, a MedDiet rich in HQ-EVOO benefitted overweight and obese patients by increasing adiponectin and IL-10 concentrations, molecules with anti-inflammatory, anti-oxidant, and insulin-sensitizing properties [69]. A decrease in oxidative stress parameters and an increase in the number of lactic acid bacteria in gut microbiota were also described. These results allowed the authors to speculate on the potential role of the MedDiet and its main component HQ-EVOO in diet-related interventions aiming to reduce inflammation and oxidative stress in overweight and obese patients [66].

The van Dijk et al. (NCT00405197) study enrolled abdominal-overweight subjects of both genders who adhered to an 8-week diet intervention. The authors tested the effect of a MedDiet rich in MUFAs from EVOO and a modified Western-type diet in which Saturated fatty acids (SFA) were substituted with EVOO MUFAs [70]. Blood samples were collected, and the levels of plasma proteins and transcriptional profiles of peripheral blood mononuclear cells (PBMC) were analyzed. The study results showed that a MedDiet high in MUFAs led to a decreased expression of oxidative phosphorylation genes, plasma connective tissue growth factor, and apoB concentrations when compared to the SFA western diet. Moreover, at the end of the study, a decrease in proinflammatory proteins was detected in the MedDiet group compared to baseline. These findings let the authors speculate that MUFA replacement could exert a beneficial effect on reducing metabolic stress and oxidative phosphorylation activity in PBMC. Again, the MedDiet seemed to lower the concentration of proinflammatory plasma proteins with a consequent antiatherogenic effect [70].

Bondia-Pons et al. (NCT01087086) published their clinical trial results based on the plasma metabolic profiling of subjects with a high BMI and at least two features of MetS [71]. The 6-month randomized controlled trial study was based on the adherence to two energy-restricted diets: the RESMENA diet (based on MedDiet key principles) and the control diet (following America Heart Association guidelines). The study was divided into two interventional time points. In the first period, the subjects received a nutritional assessment every 15 days for 2 months. Thereafter, participants carried out the habits previously learned during a 4-month self-control period. The trial aimed to analyze the short- and long-term effects of a MedDiet energy-restricted strategy on anthropometric and clinical variables. The authors used a dependency network analysis to assess associations between metabolic variables and dietary patterns. Their data evidenced changes in the plasma metabolic profiles due to adherence to the RESMENA diet with respect to the control diet at the 2-month intervention. Of note, plasmalogen phosphatidylcholine (P-18:1/20:3) and hypaphorine were identified by a dependency network analysis and were associated with reducing inflammatory markers. 

Nevertheless, these associations were lost at the study’s conclusion because of the reduced adherence to dietary patterns during the self-control period. Thus, the authors could point out how adherence to a healthy dietary pattern is a key aspect of interventional studies, and this could be a long-term challenge in populations at high risk of MetS [71]. Mathew et al. investigated the consequence of a 12-week therapeutic lifestyle change in 25 patients suffering from MetS (NCT00907127) [72]. In particular, the authors characterized the effect of MedDiet adherence and exercise on cholesterol efflux capacity (CEC), Phagocyte-derived myeloperoxidase (MPO)-mediated oxidation, and HDL proteomic profile. At the end of the 12-week study, most of the MetS components improved. In particular, CEC increased while HDL MPO oxidation products (i.e., 3-chlorotyrosine and 3-nitrotyrosine) decreased. Of note, the combinatory effect of the MedDiet and exercise contributed to the decrease of specific markers of MPO activity and improved the CEC of apoB-depleted plasma. With these results, the authors concluded that the proposed therapeutic lifestyle changes improved HDL function by inhibiting MPO-mediated oxidative stress [72]. 

Fernemark et al. carried out an interesting trial (NCT01522157) that studied the postprandial effects of three diets in type 2 diabetes patients [73]. The authors assessed the changes in glucose, insulin, and blood lipids of patients following three different dietary patterns: a low-fat diet (45–56 energy-% from carbohydrates), a low-carbohydrate diet (16–24 energy-% from carbohydrates), and a Mediterranean-style diet (32–35 energy-% from carbohydrates). Each patient experienced all three diets in randomized order. The study showed an increase in insulin response following a Mediterranean-style diet with respect to a low-fat diet, while postprandial glucose levels were comparable. Of note, this pronounced insulin increase allowed the MedDiet to maintain glucose levels similar to the low-fat meal despite the almost double calorie content. These results claimed that the caloric intake of a Mediterranean-style lunch might provide an advantageous metabolic perspective for type 2 diabetes patients [73]. 

Fortin et al. (NCT02821585) tested the differences in waist circumference, anthropometric, and metabolic outcomes in patients with both type 1 diabetes (T1D) and MetS, which were randomized into two groups: the MedDiet or low-fat diet [74]. Their results showed that both dietary patterns contribute to weight management. The study was carried out for 6 months, and the authors suggested that a long-term study could provide effective benefits of these diets [74].

Another study investigated the long-term effect of the MedDiet compared with a low-fat diet in a cohort of T2DM patients free of cardiovascular disease to assess differences in circulating levels of endothelial progenitor cells (EPCs) (NCT00725257) [75]. The authors tested whether a reduction in EPCs in T2DM patients could result from defective vascular repair and thus be linked to an increase in CVD risk. Their analysis revealed that at the end of treatment (4 years), T2DM patients adhering to a MedDiet showed an increase in circulating EPCs levels. This let the authors speculate on the effect of a MedDiet on preventing subclinical atherosclerosis progression in patients with newly diagnosed type 2 diabetes [75]. 

In summary, the findings of these trials showed the beneficial effects of the MedDiet on metabolic disorders that could be mainly related to its anti-inflammatory and anti-oxidant properties. Moreover, strict and long-lasting adherence to a MedDiet seems to be essential for its interventional improvements. 

### 5.2. The MedDiet and Cardiovascular Diseases 

The MedDiet is a widely acknowledged diet for cardiovascular health, given its effect in reducing the burden or even preventing CVD development [76]. The pioneering study providing the first evidence of the beneficial effect of the MedDiet on CVD was the Seven Country Study conducted by Keys et al. [6,77]. The authors described the incidence and the mortality due to CVD in 14 areas of seven countries belonging to the Mediterranean region. Over the years, increasing evidence has supported this initial conclusion; thus, the benefit of the MedDiet has reached a global echo [3,9,78,79]. The Lyon Diet Heart Study, a randomized secondary prevention trial aimed at testing the preventive role of Mediterranean type diet for recurrence after a first myocardial infarction, showed that protective effect of the MedDiet was maintained up to 4 years after the first infarction [80]. The MedDiet’s health power lies on the foods’ heterogeneity, allowing the proper intake of key molecules, such as long-chain polyunsaturated fatty acids, fiber, antioxidant vitamins, carotenoids, and polyphenols. Of note, this is associated with beneficial effects on endothelial and cardiovascular function [81]. Estruch et al. showed in prospective randomized controlled clinical trial enrolling high-risk patients for CVDs the decreased incidence of cardiovascular events in patients assigned to a Mediterranean Diet supplemented with extra-virgin olive oil or nuts when compared with those assigned to a reduced-fat diet [82]. The synergism between cardioprotective nutrients and MedDiet foods seems to trigger the mechanisms accountable for reducing CVD risk. These mechanisms include lowering blood pressure, lipids, endothelial dysfunction, BMI, and waist circumference and increasing the bioavailability of nitric oxide (NO), antioxidant properties, and anti-inflammatory effects [83].

Despite this evidence, many aspects related to the effectiveness of the MedDiet on CVD need to be clarified. Findings demonstrating whether the MedDiet’s beneficial effects on CVD come from its constituents or their aggregation are examples of the issues that need to be better addressed [76]. The evidence from clinical trials could accomplish these demands.

In this section, we report the results of published studies among those categorized in the Cardiovascular Diseases group providing insights into the MedDiet’s effects on CVD. 

Yubero–Serrano et al. reported on evidence from the CORonary Diet Intervention with Olive oil and cardiovascular PREVention (CORDIOPREV) study (NCT00924937) [84,85]. CORDIOPREV was a prospective trial in a cohort of 1002 coronary heart disease (CHD) patients, primarily comparing two healthy dietary patterns: The MedDiet and the low-fat diet. In particular, the authors focused on the effects of these diets on endothelial function in 805 randomized participants at baseline and after one year. MedDiet CHD patients showed an increase in the brachial artery’s flow-mediated dilation (FMD) and the endothelial progenitor cells’ content when compared to the low-fat diet group. On the other hand, the MedDiet decreased endothelial microparticle levels, whose increase is associated with endothelial injury and dysfunction [86]. This suggested a modulatory effect of the MedDiet on endothelial function. 

Moreover, a subset of 24 patients was identified to evaluate differences between the two dietary patterns in the extent of cellular processes related to endothelial damage and repair in vitro. In particular, the authors evidenced that adherence to the MedDiet resulted in low levels of intracellular ROS, apoptosis, and senescence. On the other hand, this dietary pattern increased cellular proliferation and angiogenesis. Overall, these findings suggested the effectiveness of the MedDiet in increasing endothelial function modulation and improving vascular homeostasis balance. Thus, the authors speculated that the MedDiet could be an effective strategy for recovering endothelial dysfunction in CHD patients [84]. 

Tomatoes are an integral part of the MedDiet and an essential source of lycopene, a bioactive compound with anti-oxidant properties [87,88]. Interestingly, Gajendragadkar et al. described the effect exerted by lycopene on the vasculature of CVD patients and healthy vaulters (HVs) [88]. In particular, 36 statin-treated CVD patients and 36 HVs were enrolled in a 2-month double-blind trial where 7 mg/die of lycopene or placebo were administered to the participants (NCT01100385). The primary outcome of the study was the evaluation of endothelial function. Endothelium-dependent vasodilatation (EDV) was measured by evaluating the forearm responses to acetylcholine arterial infusions through venous plethysmography. Analogously, endothelium-independent vasodilatation (EIDV) and basal NO synthase activity were measured by assessing the sodium nitroprusside and NG-monomethyl-L-arginine responses, respectively. Other biochemical and vascular endpoints were also detected, such as arterial stiffness, blood pressure, oxidized low-density lipoprotein (ox-LDL), high sensitivity C-reactive protein (hsCRP), and cytokine profile. The results showed that in CVD patients, lycopene administration improved EDV without modification in EIDV and NO responses. Of note, the post-therapy EDV response of CVD patients showed similar values to those registered in HVs at baseline. On the other hand, no differences in blood pressure, arterial stiffness, lipids, and hsCRP levels were observed in the lycopene and placebo groups of CVD and HV arms. With these results, the authors concluded that lycopene improved endothelial function in CVD patients but not in age-matched HVs. This study proves the beneficial effect of a vital component of the MedDiet on vasculature, demonstrating the need for a healthy diet to improve endothelial function in at-risk people [88].

Bédard et al. focused on sex-related differences in cardiovascular responses to diet, another key aspect of the MedDiet’s healthy effect [89]. The MedDiet’s interference with endogenous estrogen concentrations in postmenopausal women challenges its cardiovascular benefits [90]. In this paper, the authors reported the evidence of the study carried out on a cohort of 38 men and 32 premenopausal women adhering to a 4-week isocaloric MedDiet (NCT01293344). The authors compared men and premenopausal women to maximize the differences due to sex hormones. The cardiovascular risk factor was calculated by two well-recognized tools, such as the Framingham risk score and metabolic syndrome criteria, that showed good effectiveness in predicting cardiovascular morbidity and mortality [91]. The study showed that the MedDiet had a beneficial impact on cardiovascular risk regardless of gender. The analysis of all the variables studied revealed no sex by time interaction. These results agreed with the authors’ previous findings that reported no gender difference in reducing LDL-cholesterol induced by the MedDiet [92]. The conclusion of this study further underscores the MedDiet’s effectiveness in reducing cardiovascular risks even in the absence of weight loss and independently of sex-related factors [89].

Sofi et al. also compared the effect of two healthy diets on overweight omnivores with a low-to-moderate cardiovascular risk profile (NCT02641834) [93]. The MedDiet and lacto-ovo vegetarian diet (VD) were the dietary patterns analyzed, each lasting 3 months with a crossover design. The study’s primary outcome evaluated the differences in body weight, BMI, and fat mass from the study’s start. At the conclusion, the differences from baseline of cardiovascular risk parameters (i.e., lipid, glycemic, oxidative stress, and inflammatory profiles) were assayed. The authors showed that both healthy diets positively affected the primary outcome parameters. The only differences were in the effectiveness of triglyceride level reduction, which was more evident in the MedDiet compared to the VD. This, in turn, demonstrated a better regulation of LDL cholesterol levels. Both diets’ oxidative stress and inflammatory profiles showed improvements in the tested parameters, although the MedDiet significantly improved IL-17 levels [93]. 

Cicero et al. investigated the effectiveness of nutraceutical supplementation to the MedDiet to improve its beneficial effect on CVD [94]. Their trial (NCT02492464) tested nutraceutical supplementation (red yeast rice, phytosterols, and L-tyrosol) in a cohort of patients with polygenic hypercholesterolemia resistant to the MedDiet. The main parameters considered for the outcomes were lipid profile, blood pressure, arterial stiffness, and endothelial function. The authors showed that an 8-week treatment induced a favorable change in total cholesterol and LDL-C in nutraceutical-treated patients vs. placebo. An improvement in the hemodynamic parameters was also registered. Overall, these results let the authors conclude that the nutraceutical formulation effectively improved the beneficial effects of the MedDiet on cardiovascular risk factors [94]. 

In summary, the evidence reported indicates that the MedDiet and its component (i.e., lycopene) appear to positively impact the recovery of endothelial dysfunction in CVD patients. The MedDiet’s anti-inflammatory and anti-oxidative potential in CVD patients and its regulatory effect on fatty acids emerge from these studies. Further insights concern the beneficial impact on cardiovascular risk regardless of gender. This intriguing aspect should be further investigated, given that controversy still exists. The adjuvant effect of nutraceutical supplementation also emerges as a possible tool to increase the beneficial effect of the MedDiet on cardiovascular risk factors.

### 5.3. The MedDiet and Cancer

Cancer is the second leading cause of mortality worldwide [95]. The complexity of this disease relies on its multifactorial nature, encompassing key processes such as oxidative stress, chronic inflammation, alteration in cell-cycle regulation, and pro-oncogenes deregulation [96,97]. Nutrition can influence some cellular and molecular features linked to cancer; thus, a healthy lifestyle can effectively prevent 30–50% of cancers [98,99]. The MedDiet seems to fulfill these requirements because of the prevalence of foods containing anti-oxidant and anti-inflammatory molecules. Legumes, fruit, nuts, fish, vegetables, and olive oil contain bioactive nutrients, which exert a protective action by counteracting cell degeneration and cancer cell proliferation [95]. Olive oil polyphenols are key molecules accountable for the MedDiet’s protective effect. Hydroxytyrosol and its parent compound oleuropein are widely studied given their anti-inflammatory, anti-oxidant, chemopreventive, and pro-apoptotic effects. Recently, other olive oil polyphenols, such as oleocanthal, provided evidence of anti-cancer properties [96]. Despite this encouraging evidence, greater efforts are needed to provide clinical findings regarding the MedDiet’s positive impact on cancer prevention and management. The latter aspect is of interest because less is known about the effect of nutrition on people diagnosed with cancer [98].

In this section, we provide the results of the studies categorized in the Cancer group and available on PubMed. 

Bruno et al. described the effect of the MedDiet on female carriers of the BRCA mutation. Mutation in BRCA1/2 exposes females to a high risk of breast and ovarian cancer [100]. The authors reported the results obtained from a multicenter prospective randomized trial (NCT03066856). A MedDiet-based intervention with moderate protein restriction was investigated for its effectiveness in reducing metabolic factors influencing BRCA penetrance. Their hypothesis comes from evidence about the impact of Insulin-like growth factor I (IGF-I), body weight, and metabolic markers of insulin resistance on BRCA penetrance [101,102]. The study was carried out on 416 women with deleterious BRCA1/2 mutations. They were randomly assigned to an intervention and a control group and were exposed to a 6-month dietary intervention. At the end of the treatment, the intervention group patients showed a significant reduction in IGF-I levels, weight, waist circumference, hip circumference, total cholesterol, and triglycerides compared to controls. From these results, the authors concluded that the MedDiet with moderate protein restriction effectively reduced IGF-I and other potential modulators of BRCA penetrance [100]. These data have provided the basis for the analysis of the MedDiet as one of the modulators of BRCA penetrance, opening intriguing opportunities for cancer management. 

Ruiz–Vozmediano et al. described another aspect of the potential application of the MedDiet in the framework of integrative oncology [103]. This approach is used to control cancer symptoms and improve patient health and quality of life. In their paper, the authors reported the results of a randomized trial (NCT04150484) carried out on 75 women who survived to stage IIA-IIB breast cancer (BC). BC patients were split into an intervention group (IG) and a control group (CG); the former group received a 6-month dietary, exercise, and mindfulness program. At the end of the study, the IG patients showed significant BMI and weight reduction with a concomitant enhancement in healthy lifestyle habits. In particular, the authors demonstrated that MedDiet adherence was greater in the IG than the CG, which was related to their weight loss. This is interesting evidence, given that overweight and obesity have been associated with higher BC risk and poor prognosis [104]. As a result, the authors concluded that implementing multimodal interventions could effectively manage of cancer symptoms [103]. In line with this evidence, Cho et al. reported the findings from a pilot trial (NCT03581630) that assessed the combined effect of the MedDiet and naltrexone/bupropion treatment (NB) on weight loss in overweight/obese BC survivors [105]. In particular, the effectiveness of dietary strategies was tested on body weight, metabolic parameters, and quality of life. BC survivors were assigned to an 8-week intervention based on the MedDiet + NB or MedDiet alone, while the non-cancer control group adhered to the MedDiet + NB. All three groups exhibited improvement in metabolic parameters and quality of life along with a decrease in body weight, BMI, and fat mass. Thus, the authors speculated that the MedDiet, alone or in combination with naltrexone/bupropion, showed a beneficial effect on BC survivors who are overweight or obese [105]. 

Finally, Rojas Gil et al.’s pilot study (NCT04215367) described the effects of the two main components of extra virgin olive oil (EVOO) on patients suffering from chronic lymphocytic leukemia (CLL) [106]. EVOO is a major component of the MedDiet, and it is accountable for the beneficial effect exerted by this dietary pattern [17]. They assayed the impact of a dietary intervention supplemented in oleocanthal and oleacein (OC/OL) EVOO. The study was divided into two dietary interventions (DI) and administrated to CLL patients (Rai stages 0-II) who did not receive any treatment. In the DI1 group, CLL patients were divided into two sub-groups which received 40 mL/day of either high OC/OL EVOO or low OC/OL EVOO for 3 months. The DI2 group started after a 9–12 month wash-out period and lasted 6 months. It consisted of 22 patients (eight from each DI1 group and six new) who were administered with high OC/OL EVOO. The authors specified that this was a pilot study mainly focused on the tolerability of the intervention with high OC/OL EVOO. Nevertheless, their DI1 data revealed a beneficial effect of high OC/OL EVOO on hematological and biochemical markers. This effect was also confirmed in the DI2 group, where high OC/OL EVOO resulted in upregulation of ccK18 and Apo1-Fas (apoptotic markers) as well as p21 (a negative regulator of the cell cycle). A decrease in Survivin (antiapoptotic factor) and Cyclin D1 (a marker of cellular proliferation) was also detected. Overall, these data posed the first evidence of the beneficial effect of high OC/OL EVOO on the improvement of CLL by exerting a proapoptotic influence on cancer cells. A multi-center larger trial must be carried out to confirm this preliminary evidence [106]. 

Overall, these findings demonstrate the effectiveness of the MedDiet in regulating aspects that expose people to cancer risk, such as potential modulators of BRCA penetrance [100]. Moreover, the beneficial impact of a healthy life approach in managing cancer symptoms in BC survivors who are overweight or obese also emerges in our examination. This is extremely encouraging, given that randomized controlled trial evidence is the gold standard for establishing the prognostic value of dietary factors. Finally, the effectiveness of OL and OC in CLL improvement also confirms the beneficial role of olive oil, the major component of the MedDiet, on cancer prevention. 

## 6. Conclusion and Perspective

We provided an update of the clinical trials in which the direct or indirect effects of the MedDiet were evaluated on health and specific diseases. The importance of the results obtained by MedDiet-based trials is well acknowledged both for clinical and social implications. Our findings revealed an increase in the number of clinical trials in the last decade that pairs with the enhanced level and quality of the evidence concerning the effects of this dietary pattern [2]. We found that the most representative diseases challenged with the MedDiet are cardiovascular diseases, metabolic diseases, and cancer. This is proof of the beneficial potential of this dietary pattern on chronic diseases affecting people worldwide. Nevertheless, a discrepancy exists between the increased number of completed clinical trials and the few published results (positive or negative). This should be better addressed since these data could be instrumental in preventing potentially inefficacious pleonastic clinical trials and providing novel evidence-driven studies. 

In summary, the studies’ findings disclose that the MedDiet’s beneficial effects could be primarily related to its anti-inflammatory and anti-oxidant properties. The effectiveness of this dietary pattern in controlling waist circumference and obesity seems to be another key aspect. Moreover, strict and long-lasting adherence to the MedDiet as well as the beneficial effects of specific components (e.g., olive oil or its polyphenols) seem to emerge as useful insights for interventional improvements. The synergic effect of the MedDiet and physical exercise has also been exanimated, mainly in cancer survivor patients. This is proof of the emerging evidence that addresses the MedDiet as a healthy lifestyle rather than a simple dietary pattern. Moreover, the intriguing interaction between MedDiet’s components and the immune system will pave the way for tailored studies aiming to dissect this complex interplay. These findings will provide further insights useful for the clinical practice. 

The MedDiet has recently emerged as a suitable model of sustainable nutrition, in which nutrition, local foods, biodiversity, culture, and sustainability are strictly interconnected. Dietary habits are drivers of environmental pressure, and it seems that the MedDiet could beneficially impact this aspect. The low environmental impact of the MedDiet with respect to other dietary patterns has been estimated regarding water consumption, nitrogen emission, and carbon footprint [2,3]. Indeed, the MedDiet is essentially a plant-based diet with few animal products, mainly red meat consumed monthly. Animal-based foods are the most land-and-energy intensive. On the other hand, vegetables, cereals, nuts, and olive oil lower GHG emissions [107].

In conclusion, this evidence demonstrates the importance of the MedDiet as the combination of a healthy dietary pattern and healthy behaviors. The conveyance of this model toward other populations and countries is desirable to export its beneficial implications both for clinical and environmental aspects. Moreover, evidence from long-term studies is needed to sustain the promising data described here and further dissect the functional implications of the MedDiet for chronic pathologies such as cardiovascular diseases, metabolic diseases, and cancer. This is a suitable approach to defining the MedDiet’s beneficial contribution to risk factors and other outcomes related to these diseases.
